# Beneficial effects of *Panax notoginseng* (Burkill) F. H. Chen flower saponins in rats with metabolic hypertension by inhibiting the activation of the renin–angiotensin–aldosterone system through complement 3

**DOI:** 10.1186/s12906-022-03828-2

**Published:** 2023-01-18

**Authors:** Qiqi Huang, Jie Su, Jie Xu, Huanhuan Yu, Xiaohu Jin, Yajun Wang, Meiqiu Yan, Jingjing Yu, Suhong Chen, Youhua Wang, Guiyuan Lv

**Affiliations:** 1 School of Pharmaceutical Sciences, Zhejiang Chinese Medical University, Hangzhou, China; 2 Collaborative Innovation Center of Yangtze River Delta Region Green Pharmaceuticals, Zhejiang University of Technology, Hangzhou, China; 3 Longhua Hospital, Shanghai University of Traditional Chinese Medicine, Shanghai, China

**Keywords:** Complement 3, Endothelial function, Metabolic hypertension, *Panax notoginseng* (Burkill) F. H. Chen flower saponins, Renin–angiotensin–aldosterone system

## Abstract

**Background:**

Metabolic hypertension (MH) has become the most common type of hypertension in recent years due to unhealthy eating habits and lifestyles of people, such as over-eating alcohol, high fat, and sugar diets (ACHFSDs). Therefore, effective means to combat MH are needed. Previous studies have shown that *Panax notoginseng* (Burkill) F. H. Chen flower saponins (PNFS) can lower blood pressure in spontaneously hypertensive rats (SHR). However, whether it acts on MH and its mechanism of action remain unclear.

**Methods:**

The pharmacodynamic effects of PNFS were evaluated in rats with ACHFSDs-induced MH. The blood pressure, blood biochemical, grip strength, face temperature, vertigo time, and liver index were estimated. The histological changes in the liver and aorta were observed using hematoxylin and eosin staining. The levels of ET-1, TXB_2_, NO, PGI_2_, Renin, ACE, Ang II, and ALD in plasma were detected using ELISA. The levels of C3, KLF5, LXRα, and Renin in kidney tissues were measured using qRT-PCR.The expression levels of C3, KLF5, LXRα, and Renin in kidney tissues were examined using Western blotting.

**Results:**

In the present study, PNFS was found to reduce blood pressure, face temperature, and vertigo time, increase grip strength and improve dyslipidemia in rats with MH. In addition, PNFS decreased the plasma levels of ET-1 and TXB_2_, elevated the levels of NO and PGI_2_, and improved pathological aortic injury. Meanwhile, PNFS decreased the plasma levels of Renin, ACE, Ang II, and ALD. QRT-PCR and Western bolt showed that PNFS downregulated C3, KLF5, LXRα, and Renin protein and mRNA expression in the kidneys of rats with MH.

**Conclusion:**

The finding of the present study suggested that PNFS could downregulate C3 and KLF-5 expression in rats with MH, thereby inhibiting the overactivation of the renin–angiotensin–aldosterone system, while improving vascular endothelial function and ultimately reducing blood pressure in rats with MH.

**Supplementary Information:**

The online version contains supplementary material available at 10.1186/s12906-022-03828-2.

## Introduction

Hypertension is one of the most common cardiovascular diseases that seriously endanger human health. According to the latest epidemiological survey on hypertension, 44.7% of Chinese people aged 35–75 years suffer from hypertension [1]. More recently, 23.2% (an estimated 244.5 million) of Chinese adults are reported to have hypertension [2]. In recent years, metabolic disorders have become a major driver of the increasing prevalence of hypertension due to the growing number of irrational dietary practices in humans (over-eating alcohol, high fat, and sugar diets (ACHFSDs) [3, 4]. According to a report [5, 6], more than 80% of patients with hypertension have different forms of metabolic abnormalities, of which 55.3% have abnormal blood glucose, 69.5% have dyslipidemia, and only 10–20% have hypertension alone. Therefore, modern scholars consider hypertension as a metabolic disease and have proposed the concept of metabolic hypertension (MH), which helps control blood pressure by improving metabolic abnormalities [7, 8].

The complement system has recently been found to be associated with metabolic disorders, including metabolic syndrome, hypertension, obesity, and other cardiovascular risk components [9]. The complement system is a specific activating protein that can mediate immune and inflammatory responses in vivo and is closely associated with cardiometabolic diseases [10, 11]. Complement 3 (C3) is a central component of the complement system, which is produced mainly by the liver, monocytes, and macrophages. Its activation leads to the initiation of the terminal complement pathway and the signal for the release of the allergenic toxin C3a [12]. C3a mediates smooth muscle contraction and increases vascular permeability by decreasing the local inflammatory response [13, 14]. A recent study has shown that C3 levels are elevated in patients with metabolic syndrome, suggesting that it may be involved in the development of metabolic syndrome [15].

In particular, a large body of epidemiology has demonstrated that C3 can be a potential predictor of cardiovascular events [16]. A clinical study found that elevated serum C3 levels were associated with prehypertension and correlated with elevated blood pressure and the development of hypertension in the future [17, 18]. Meanwhile, another study showed that right ventricular systolic pressure and right ventricular hypertrophy are attenuated in C3–/– hypoxic mice, suggesting that C3 deficiency attenuates chronic hypoxia-induced pulmonary arterial hypertension in mice [19]. C3 induces the phosphorylation of extracellular signal−regulated kinase in vascular smooth muscle and increases Krüppel-like factor 5 (KLF5) promoter activity [20]. KLF5-specific inhibitors suppress C3a-induced increase in renin mRNA expression in epithelial cells [21]. This suggests that KLF5 is a key transcription factor in C3-induced renin production. In vitro, Liver X receptor α (LXRα) binds non-canonical response elements in the renin promoter and regulates renin transcription, and C3a enhances nuclear staining for LXRα and increases renin expression in TCMK-1 cells [21, 22].

Renin and Ang II are essential components of the renin–angiotensin–aldosterone system (RAAS), which regulates blood pressure and maintains water–electrolyte balance to maintain the relative stability of the body’s environment [23]. Renal secretion of angiotensin-converting enzyme (ACE) acts on its substrate to form Ang II. There is an increase in renin activity which leads to an increase in Ang II, activation of the RAAS and results in hypertension, cell proliferation, inflammation, and fibrosis. This suggests that it may be possible to induce RAAS alterations via C3, indicating a potential strategy to improve MH.

MH is a disease with a high prevalence and low treatment rate, which urgently requires a medical breakthrough. Traditional Chinese medicine has played a vital role in the health care of the people in China. It is recognized worldwide as a powerful alternative for treating cardiovascular diseases [24]. *Panax notoginseng* (Burkill) F. H. Chen, a well-known and valuable traditional Chinese medicine, displays a superior protective effect on cerebrovascular diseases [25]. Saponin is considered to be the main active ingredient in *P. notoginseng* (Burkill) F. H. Chen and is deemed to be a promising drug against many chronic diseases, especially cardiovascular and hematological diseases [26–28]. Among the different parts of *P. notoginseng* (Burkill) F. H. Chen, flowers are rich in saponins [29, 30]. In everyday life, flower buds are often consumed as a tea to fight hypertension, calm the liver, and improve vision [31]. Modern pharmacological research has shown that the *P. notoginseng* (Burkill) F. H. Chen flower saponins (PNFS) have antihypertensive, vascular repair, and heart- and liver-protective effects [32–35]. A previous study found that PNFS steadily lowered the blood pressure of spontaneously hypertensive rats (SHR) [36, 37]. However, whether PNFS also modulates ACHFSDs-induced MH is not clear.

Therefore, in the present study, ACHFSDs-induced MH rats were treated with PNFS to assess the changes in blood pressure, lipids, behavior, histopathology, C3, and RAAS to investigate the hypotensive effects of PNFS in rats with MH and its possible mechanisms.

## Methods

### Chemicals and reagents

The standard reagents of total cholesterol (TC), triglyceride (TG), low-density lipoprotein cholesterol (LDL-c), and high-density lipoprotein cholesterol (HDL-c) were purchased from Ningbo Medical System Biotechnology Co., LTD (Ningbo, Zhejiang, China). The antibodies against C3 (Cat#ab200999, Lot#GR3354270-3), KLF5 (Cat#ab24331, Lot#GR3236297-14), LXRα (Cat#ab106464, Lot#GR135377-8), and GAPDH (Cat#ab181602, Lot#GR3285728-3) were obtained from Abcam (Cambridge, MA, USA). The antibodies against Renin (Cat#sc-133,145, Lot#A0722) were purchased from Santa Cruz Biotechnology, INC. (Santa Cruz, CA, USA). Trizol® Plus RNA Purification Kit, SuperScript™ III First-Strand Synthesis Supermix for qRT-PCR were purchased from Invitrogen (Carlsbad, CA, USA). Endothelin 1 (ET-1, Cat#MM-0560R1), thromboxane B_2_ (TXB_2_, Cat#MM-0516R1), nitric oxide (NO, Cat#MM-20607R1), prostaglandin I_2_ (PGI_2_, Cat#MM-0793R1), Renin (Cat#MM-0343R1), ACE (Cat#MM-0212R1), Ang II (Cat#MM-0211R1), and aldosterone (ALD, Cat#MM-0555R1) detection kits were obtained from Jiangsu Enzyme Industrial Co., Ltd (Yancheng, Jiangsu, China).

### Preparation of PNFS

**Fig. 1:**
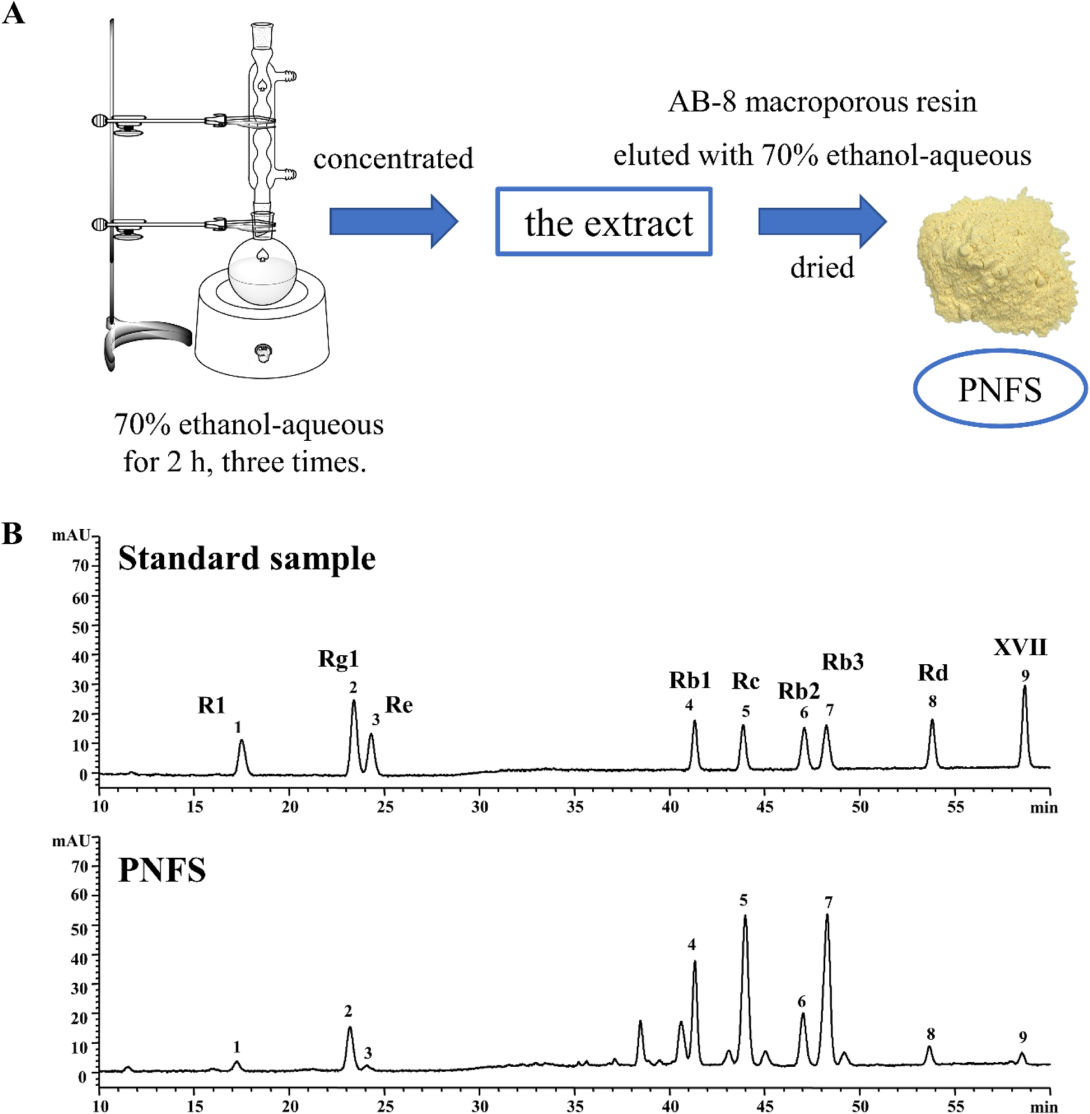
Flow chart of *Panax notoginseng* (Burkill) F. H. Chen flower saponins (PNFS) extraction and determination. **A** Extraction flowchart of PNFS. **B** High-performance liquid chromatography (HPLC) analysis of PNFS

The flowers of *P. notoginseng* (Burkill) F. H. Chen were collected from Wenshan, Yunnan Province, China, and identified by Professor Wei Li of the School of Pharmacy, Shanghai University of Traditional Chinese Medicine. To obtain saponin-rich PNFS, we extracted 1 kg of flowers of *P. notoginseng* (Burkill) F. H. Chen with 14 volumes of 70% ethanol-aqueous solution at reflux for 2 h. This process was performed three times and combined all the liquids. It was then concentrated on a rotary evaporator to approximately 0.4 g/mL. The concentrate was loaded on an AB-8 macroporous resin column according to the previous method [38] and eluted with three column volumes of 70% ethanol-aqueous at a flow rate of two-column volumes per hour. After drying, PNFS dry powder was obtained. The process is shown in Fig. 1A.

### Analysis of saponins in PNFS

The saponins in PNFS were determined by an HPLC (1,200 series, Agilent Technologies). The chromatographic conditions are as follows: chromatographic column: Agilent Zorbax Eclipse XDB-C18 (250 mm × 4.6 mm, 5 μm); mobile phase (A: acetonitrile, B: water). The gradient elution conditions are as follows: 0 ~ 10 min, 20%~20%A; 10 ~ 25 min, 20%~22%A; 25 ~ 30 min, 22%~30%A; 30 ~ 45 min, 30%~32%A; 45 ~ 70 min, 32%~40%A; 70 ~ 80 min, 40%~60%A. The flow rate: 1.0 mL/min; column temperature: 25 ℃; detection wavelength: 203 nm; injection volume: 10 µL. Saponins in PNFS contain as following: ginsenoside Rc (38.94%), ginsenoside Rb2 (12.36%), ginsenoside Rb1 (10.97%), ginsenoside Rg1 (8.31%), ginsenoside Rb3 (6.62%), ginsenoside Rd (4.14%), notoginsenoside R1 (3.44%), gypenoside XVII (1.33%), and ginsenoside Re (0.97%). High-performance liquid chromatography (HPLC) analysis of PNFS is shown in Fig. 1B.

### Animals and treatment

**Fig. 2:**
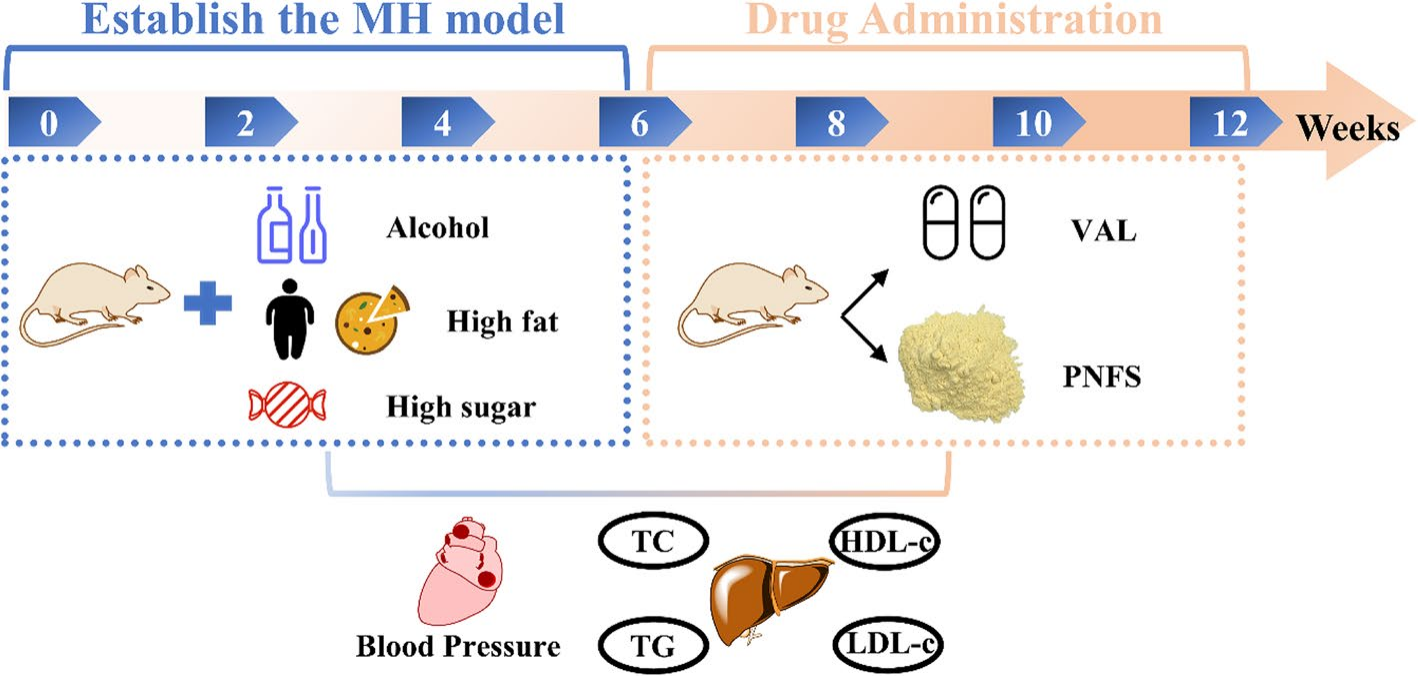
The experimental design

Sixty male Sprague Dawley rats, weighing 180–220 g, were maintained on a standard laboratory diet and exposed to a 12-hour/12-hour light-dark cycle at 23 ± 2 ℃ (Zhejiang Academy of Medical Sciences, animal certification number: No. 2,005,190,007). All rats were allowed to eat standard food and drink water freely for one week before using for the experiment. Ten rats were randomly selected as the normal control group (NC) according to the random number table. The remaining rats were used for modeling and were fed ACHFSDs, which consisted of high-fat/high-sugar feed (TROPHIC Animal Feed High-Tech Co. Ltd) and alcohol (In the first week, the alcohol concentration was gradually increased from 5 to 10%. In the next 5 weeks, the alcohol concentration was increased by 2% per week until 20%.). The composition of the MH diet in this study was 20.0% sucrose, 15% lard, 0.8% cholesterol, 0.2% sodium cholate, and 64% standard diet. The type of alcohol is commercially available 52° Chinese Baijiu (Red Star Company, Beijing, China). During this time we tested blood pressure, TG, TC, LDL-c, and HDL-c. Approximately 6 weeks later, 46 rats with a systolic blood pressure higher than 140 mmHg were randomly divided into the model control group (MC, pure water, *n* = 10), Valsartan group (VAL, 8.0 mg/kg, *n* = 9), PNFS low dose group (PNFS-L, 30.0 mg/kg, *n* = 9), PNFS middle dose group (PNFS-M, 60.0 mg/kg, *n* = 9), and PNFS high dose group (PNFS-H, 120.0 mg/kg, *n* = 9) via oral daily gavage for six weeks (the entire extract of PNFS). Observe the animals’ body condition daily to ensure the animals’ health during the experiment. Animal protocols were approved by the Zhejiang Chinese Medical University of Science Animal Care and Welfare Committee (permit number: ZSLL-2017-185). The experimental design is shown in Fig. 2.

### Blood pressure measurement

Measurements of blood pressure in rats were performed using a tail-cuff method (Softron, Tokyo, Japan). For blood pressure monitor adaptation, rats were adaptively trained before starting measurements. After adaptive training, the monitor was used to obtain stable systolic blood pressure (SBP), diastolic blood pressure (DBP), and mean arterial blood pressure (MBP).

### Blood biochemical measurement

Blood samples were obtained at 6, 8, and 10 weeks by capillary puncture of the orbital venous plexus. The serum samples obtained after centrifugation (3,500 r × 15 min). TC, TG, HDL-c, and LDL-c levels in rat serum were measured using a TBA-40FR full automated biochemical analyzer (Toshiba, Japan).

### Grip strength test

YLS-13 A grip strength apparatus (Jinan, China) was used to determine the maximum force generated when a rat could not hold the grip plate. When the rat grasped the grasping board, pulled its tail horizontally backwards. Grip Strength, the force applied to the plate just before rats lost its grip. Rats were pulled three times and the average of the three times was used to calculate the grip strength.

### Face temperature measurement

The temperature of the rat’s face was measured in a quiet environment using an infrared non-contact thermometer. Each rat was measured three times, and the average value was used for statistical analysis.

### Balance rotarod test

ZH-SZ balance rotator (Anhui, China) was used to gauge the balance of rats. Rats were placed in the rotating cage and the machine was initiated with 800 r/min. Stop after one minute, the time needed for the rats to return to normality was recorded as vertigo time.

### Enzyme-linked immunosorbent assay

Blood samples were collected from the abdominal aorta after deep anesthesia of rats by intraperitoneal injection of sodium pentobarbital (50 mg/kg) at the 12th week. The plasma samples were obtained by centrifugation (3,500 r × 15 min). The levels of ET-1, TXB_2_, NO, PGI_2_, Renin, ACE, Ang II, and ALD were measured by enzyme-linked immunosorbent assay kit, according to the manufacturer’s requirements.

### Liver index

Before anesthesia, the rats were weighed, and subsequently, the livers were removed and weighed. Liver index = liver weight (g)/body weight (g) × 100%.

### Hematoxylin and eosin (H&E) staining

The liver and aorta tissue of each rat was fixed with 10% neutral-buffered formalin for 2 days, followed by graded dehydration with alcohol from a concentration of 50–100%, wax immersion, paraffin embedding, and sectioning. After obtaining 4 μm paraffin sections, the sections were then dewaxed, stained with hematoxylin for 5 min and eosin for 3 min, then sealed with neutral resin and dried to observe histomorphological changes under light microscopy.

### Quantitative real-time PCR

Kidneys were collected 12 h after the last drug treatment. After being washed with saline, the tissue samples were blotted dry and collected in lyophilized tubes, and first placed in liquid nitrogen for snap freezing, after which they were stored at -80 °C. Real-time PCR was used to determine the mRNA expression of C3, KLF-5, LXRα, and Renin. Primer sequences are in Table 1. The specific operation is as follows: RNA extraction from tissues was performed using Trizol reagent (Invitrogen, Carlsbad, CA, USA) according to the kidney and reverse transcribed into cDNA. Finally, the fluorescence signal was measured using multiple Real-time fluorescence quantitative PCR machines (Bio-Rad CFX384, USA). The reaction conditions of Real-Time PCR: 95 ℃ for 1 min, followed by 40 PCR cycles, each cycle consisting of 95 ℃ for 15 s, 63 ℃ for 25 s. We used GAPDH as an internal reference, and each sample was repeated three times. The relative expression value was calculated with the formula: △Ct = Ct (target gene) - Ct (reference gene), the relative = 2^−△△Ct^.

**Table 1:** Real-time PCR primers sequences

Gene	Genbank Accession	Primer Sequences (5’to3’)
GAPDH	NM_017008.4	GAAGGTCGGTGTGAACGGATTTG
		CATGTAGACCATGTAGTTGAGGTCA
LXRα	NM_031627.2	CTGATGTTCCCACGGATGCTAAT
		CCAACACAGAGGACACGGAGAA
C3	NM_016994.2	ACGTCAGGGTCCCAGCTA
		CCGCAGGACATTGGGAGTAA
KLF-5	NM_053394.3	TCCTATGCTGCTACAATTGCTTCGA
		CCGGGTTACTCCTTCTGTTGT
Renin	J02941.1	CTCTTGTTGCTCTGGACCTCTT
		CGCTCCTCCAGGATTTCC

### Western blot analysis

The kidney tissues were extracted using a total protein extraction kit, and total protein was quantified using an Enhanced BCA Protein Assay Kit. 10% sodium dodecyl sulfate-polyacrylamide gel electrophoresis was used to separate proteins, which were electro-transferred to polyvinylidene fluoride membranes. After blocking with T-TBS that contained 5% BSA for 1 h, the membranes were incubated with various specific primary antibodies, namely, anti-C3, anti-KLF-5, anti-LXRα, anti-Renin, overnight at 4 ℃. After that, the membranes were washed with T-TBS three times, then incubated with secondary antibodies for 1 h at room temperature. Antibody binding was detected with ECL kit (GE Healthcare). Quantification of expression levels was performed by using the ImageJ software (NIH, Bethesda, MD, USA).

### Statistical analysis

Results are expressed as mean ± SEM. Multiple groups were compared using a One-way ANOVA with LSD as a post hoc comparison (IBM SPSS Statistics 20, SPSS Inc., IL, USA). Differences were considered significant at *p* < 0.05. Graphing was done using GraphPad Prism 8.

## Results

### PNFS decreased blood pressure levels in rats with MH

**Fig. 3:**
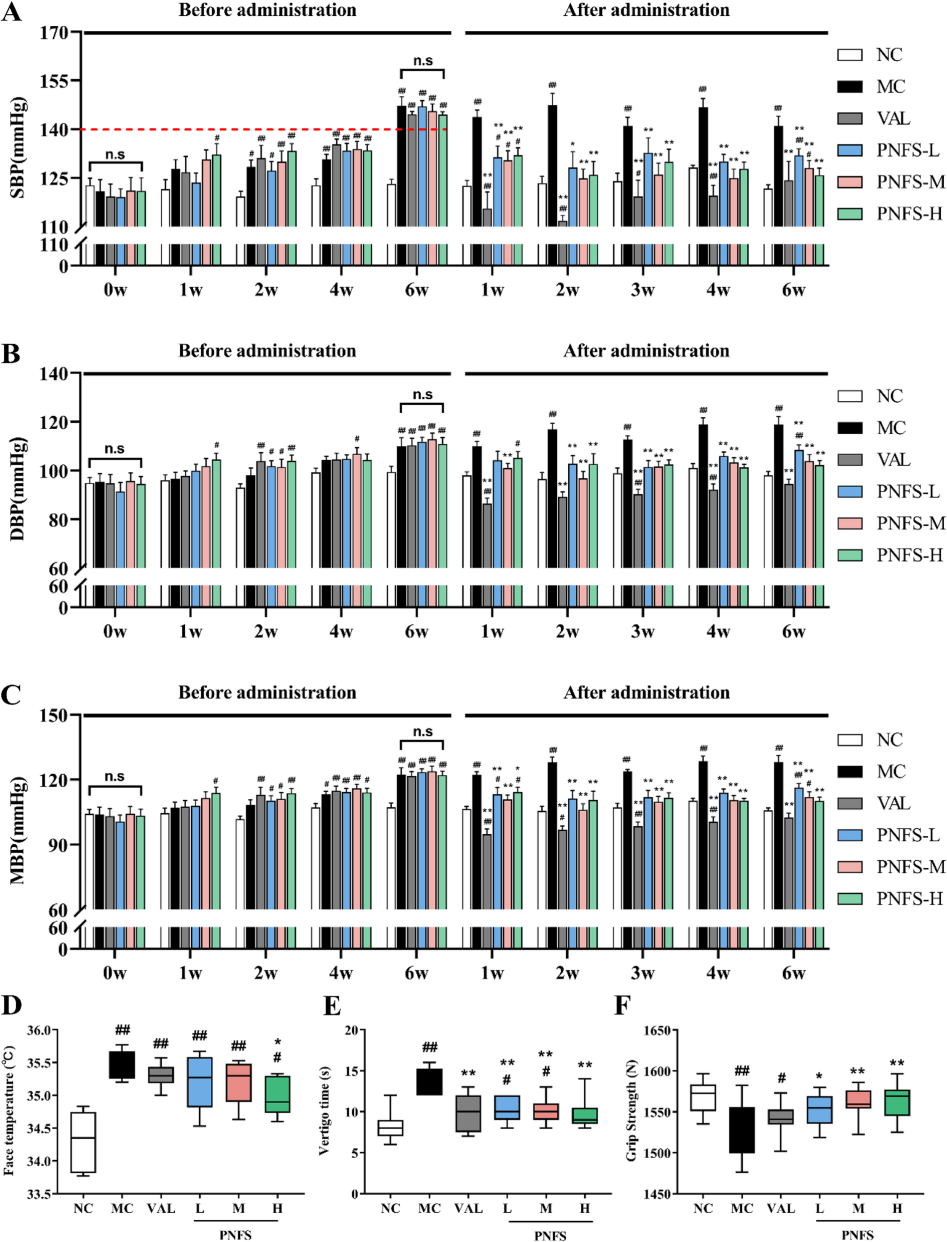
Effect of PNFS on behavioral signs in MH rats. **A** Changes in SBP. **B** Changes in DBP. **C** Changes in MBP. **D** Changes in face temperature. **E** Changes in vertigo time. **F** Changes in grip strength. NC: normal control group; MC: model control group; VAL: valsartan group; PNFS-L: PNFS low dose group; PNFS-M: PNFS middle dose group; PNFS-H: PNFS high dose group. ^#^*P* < 0.05, ^##^*P* < 0.01 vs. the normal control group; ^*^P < 0.05, ^**^P < 0.01 vs. the modle control group, *n* = 8–10

The SBP, DBP, and MBP of MH rats started to rise gradually from 2 weeks after modeling. By 6 weeks after modeling, significant differences were found compared with the NC (*P* < 0.01), and the SBP could reach 140 mmHg or more, indicating successful modeling. Compared with the MC, SBP, DBP, and MBP of rats in the PNFS-L, PNFS-M, and PNFS-H groups were significantly lowered after 2 weeks of administration (*P* < 0.01 and 0.05), and the blood pressure could be lowered stably and continuously until 6 weeks. It indicated that high-sugar and high-fat combination drinking could raise blood pressure in rats, and PNFS could stably lower blood pressure in these rats (Fig. 3A–C).

### PNFS reduced face temperature and vertigo time and increased grip strength in rats with MH

As shown in Fig. 3D–F, compared with the NC, the rats with MH had significantly higher face temperature and vertigo time and significantly lower grip strength (*P* < 0.01). PNFS significantly increased grip strength and decreased vertigo time (*P* < 0.01 and 0.05), and PNFS-H significantly decreased face temperature (*P* < 0.05).

### PNFS reduced blood lipid levels and attenuated the hepatic steatosis in rats with MH

**Fig. 4:**
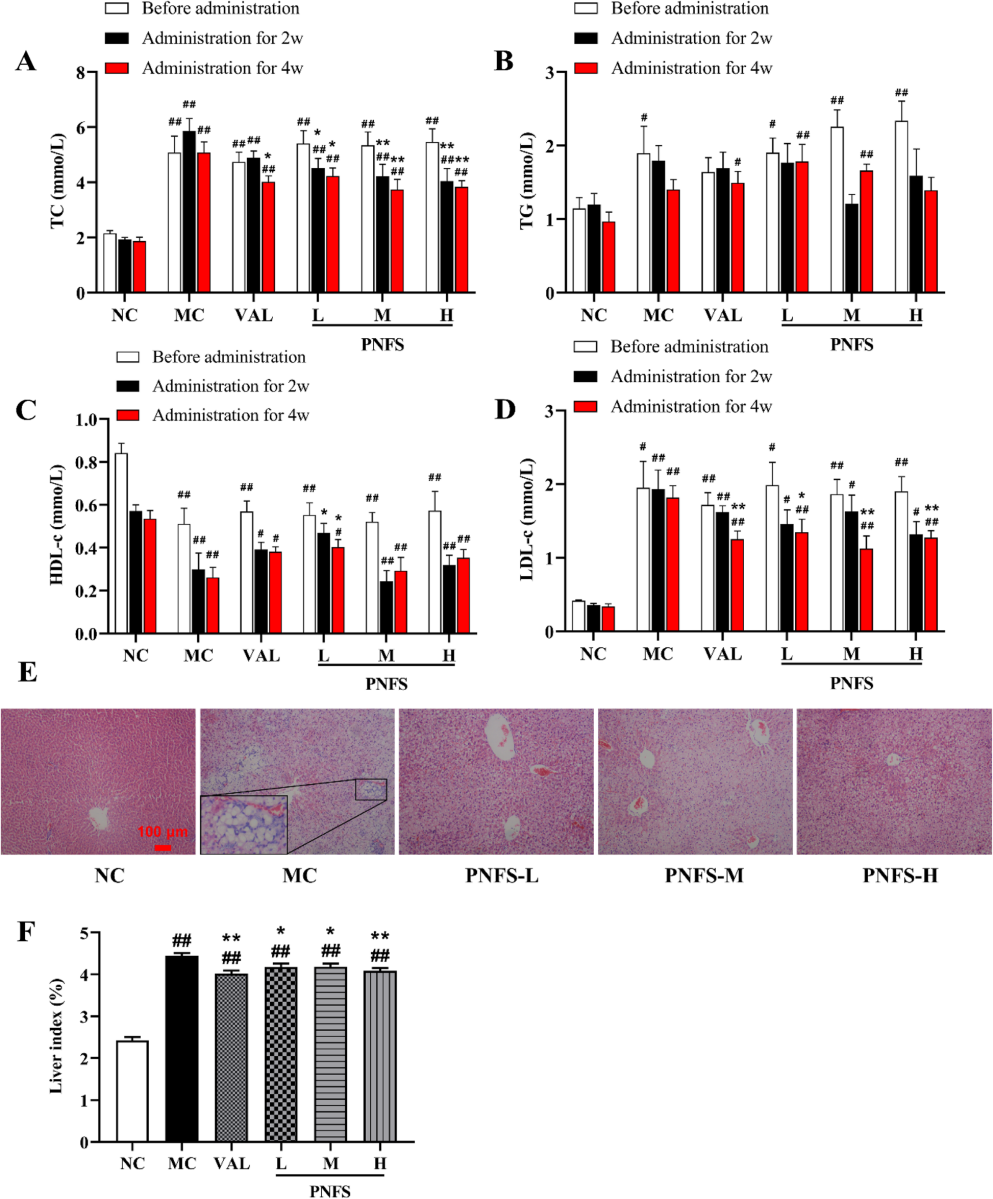
Effect of PNFS on blood lipids and liver of MH rats. **A** Changes in total cholesterol (TC). *n* = 7. **B** Changes in triglyceride (TG). *n* = 7. **C** Changes in high-density lipoprotein cholesterol (HDL-c). *n* = 7. **D** Changes in low-density lipoprotein cholesterol (LDL-c). *n* = 7. **E** Representative photomicrographs of liver tissue sections stained with H&E (100 ×). **F** Changes in liver index. *n* = 9–10. ^#^*P* < 0.05, ^##^*P* < 0.01 vs. the normal control group; ^*^P < 0.05, ^**^P < 0.01 vs. the modle control group

After 6 weeks of modeling, the levels of TC, TG, and LDL-c significantly increased (*P* < 0.01 and 0.05) and the level of HDL-c significantly decreased (*P* < 0.01) in rats with MH compared with the NC. After 2 weeks of administration, the TC level significantly decreased (*P* < 0.01 and 0.05) in the PNFS group and the HDL-c level significantly increased in the PNFS-L group (*P* < 0.05) compared with the MC. After 4 weeks of administration, the levels of TC and LDL-c were significantly lower in the PNFS group (*P* < 0.01 and 0.05), and the improvement effect was better than that after 2 weeks. The HDL-c level was significantly higher in the PNFS-L group (*P* < 0.05), but no statistically significant difference was found in the TG levels (Fig. 4A–D).

The H&E staining results also showed abnormal liver in rats with MH. We observed that the hepatocytes of normal control rats were neatly arranged, with abundant cytoplasm and intact and clear nuclei; the hepatocytes of rats with MH were significantly enlarged, with a large number of fat vacuoles visible in the cytoplasm and diffuse steatosis of hepatocytes; and the hepatocytes in the PNFS group rats were less swollen and had significantly fewer fat vacuoles (Fig. 4E). Meanwhile, we also found that the liver index of rats with MH increased significantly (*P* < 0.01), and PNFS significantly decreased the liver index (Fig. 4F) (*P* < 0.01 and 0.05). Taken together, we suggested that PNFS could improve abnormal lipid metabolism and hepatic steatosis to some extent.

### PNFS improved vascular endothelial function in rats with MH

**Fig. 5:**
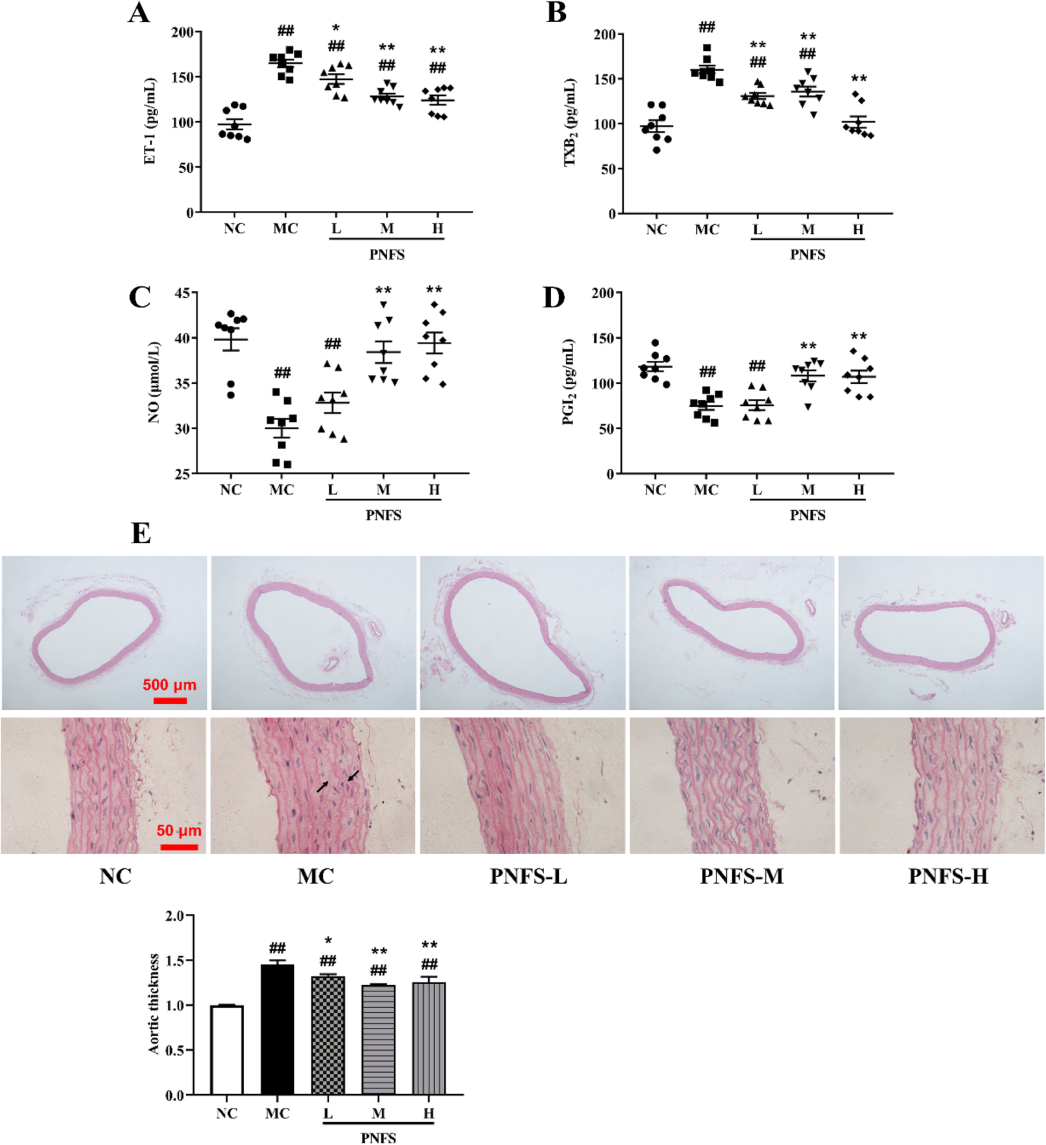
Effect of PNFS on vascular endothelium of MH rats. **A** Changes in endothelin 1 (ET-1). **B** Changes in thromboxane B_2_ (TXB_2_). **C** Changes in nitric oxide (NO). **D** Changes in prostaglandin I_2_ (PGI_2_). **E** Representative photomicrographs of aorta tissue sections stained with H&E (40 ×, 400×). The black arrows represent tissue lesions. ^#^*P* < 0.05, ^##^*P* < 0.01 vs. the normal control group; ^*^P < 0.05, ^**^P < 0.01 vs. the model control group, *n* = 8

The endothelium controls vascular tone by releasing vasoconstrictors such as ET-1, TXB_2_, and vasodilators such as NO and PGI_2_ [39]. Compared with the NC, rats with MH showed significantly higher levels of ET-1 and TXB_2_ (*P* < 0.01) and lower levels of NO and PGI_2_ (*P* < 0.01), indicating an impaired endothelial function in MH rats. PNFS administration significantly decreased the levels of ET-1 (*P* < 0.01 and 0.05) and TXB_2_ (*P* < 0.01) and increased the levels of NO and PGI_2_ (*P* < 0.01) (Fig. 5A–D).

The H&E staining results also showed that the aorta of normal control rats was structurally normal. In contrast, the aorta of rats with MH had thickened intima, hypertrophied, and disorganized vascular smooth muscle cells, and misaligned, interrupted, and detached endothelial cells. However, treatment with PNFS significantly inhibited the high-sugar and high-fat combination alcohol-induced histopathological changes in the aorta tissues. (Fig. 5E). These results indicated that PNFS could effectively improve vascular endothelial function and aortic lesions in rats with MH.

## PNFS reduced C3 levels and inhibited RAAS activation

**Fig. 6:**
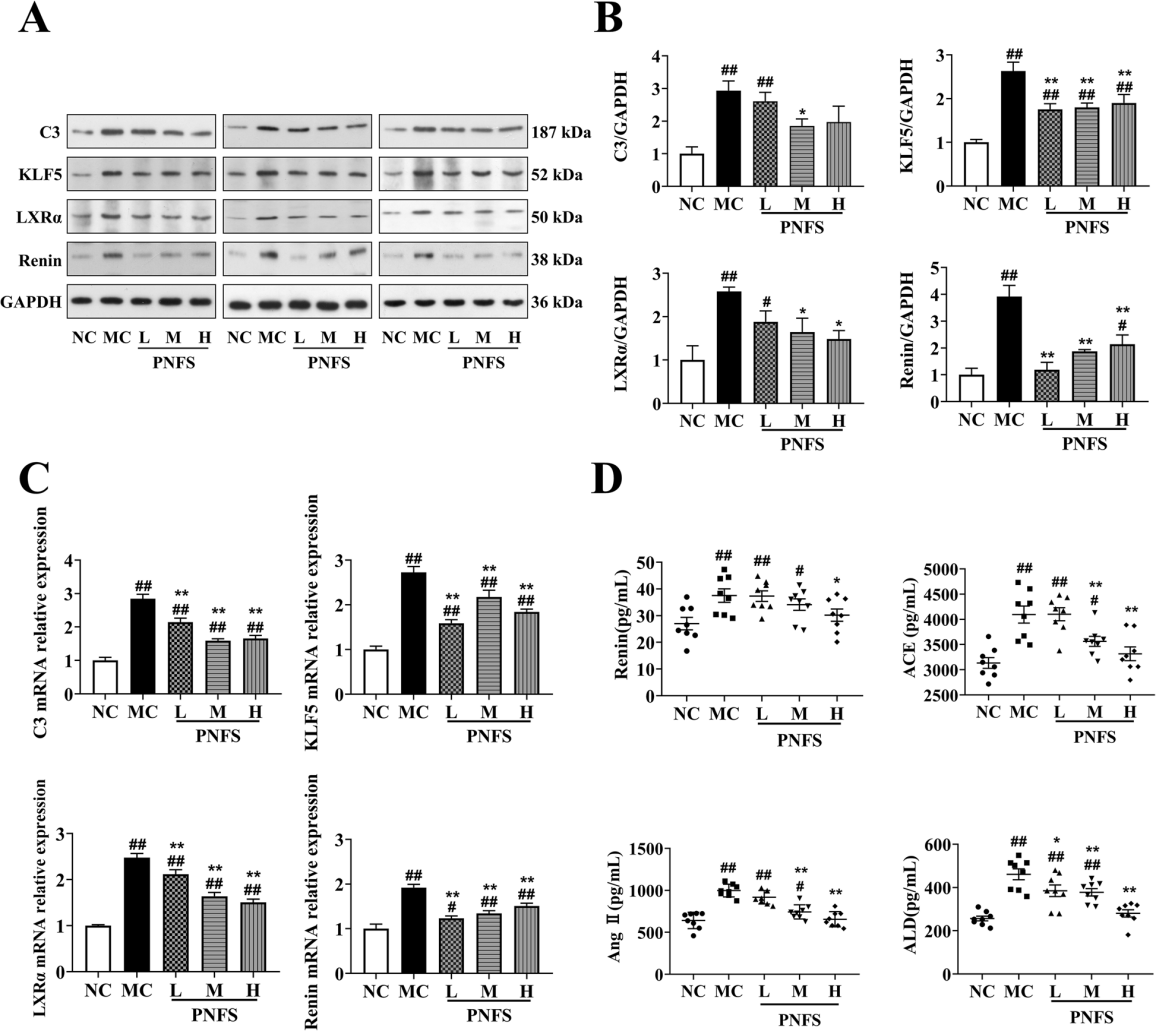
Effect of PNFS on C3/RAAS of MH rats. **A** Western blotting bands of C3, KLF5, LXRα, and Renin in kidney tissue of rats. **B** Gray intensity analysis. *n* = 3. **C** Relative expressions of C3, KLF5, LXRα, and Renin mRNA. *n* = 3. **D** Changes in Renin, ACE, Ang II, and ALD. n = 8. ^#^*P* < 0.05, ^##^*P* < 0.01 vs. the normal control group; ^*^P < 0.05, ^**^P < 0.01 vs. the modle control group

WB and qPCR results showed that compared with the NC, the protein and mRNA expression of C3, KLF5, LXRα, and Renin significantly increased (*P* < 0.01), and PNFS could significantly reduce the protein and mRNA expression of C3, KLF5, LXRα and Renin in the kidney of rats with MH (*P* < 0.01 and 0.5) (Fig. 6A–C).

Compared with the NC, the plasma levels of Renin, ACE, Ang II, and ALD were significantly increased (*P* < 0.01) and the plasma levels of Renin, ACE, Ang II, and ALD were decreased by PNFS administration in MH rats (*P* < 0.01 and 0.5) (Fig. 6D). The results suggested that PNFS could effectively reduce C3 levels and inhibit RAAS activation.

## Discussion

MH is characterized by multiple metabolic disorders, often leading to cardiovascular and cerebrovascular complications, and the pathological mechanisms behind its development are complex [4]. Some scholars suggested that irrational dietary habits are responsible for the initiation of MH [8]. Therefore, we established an MH rat model by ACHFSDs induction, which mimicked irrational dietary habits in humans. We found that rats with MH exhibited elevated blood pressure, elevated face temperature, elevated vertigo time, reduced grip strength, dyslipidemia, and hepatic steatosis, which were similar to those in patients with high blood pressure, irritability, dizziness, fatigue, high blood lipid levels, and liver impairment [40–42].

To change these manifestations, we considered *Panax notoginseng* (Burkill) F. H. Chen flower, which is often used as a tea to fight hypertension and sedate the liver [31]. Saponins are the most important active ingredients in *P. notoginseng* (Burkill) F. H. Chen flower [29, 30]. In the present study, we detected that PNFS contained nine saponins, of which the more abundant ginsenoside Rb1, ginsenoside Rg1, ginsenoside Rb3 and ginsenoside Rd all clearly have certain anti-hypertensive effects [43–46], which may be the basis of the anti-hypertensive components of PNFS, while the most abundant ginsenoside Rc and ginsenoside Rb2 are currently reported to have mainly cardioprotective effects [47, 48], and whether they have anti-hypertensive effects remains to be further investigated. Previous studies have shown that PNFS can lower blood pressure in SHR, reduce the levels of TC and LDL-c, and regulate lipids in rat hyperlipidemia models [36, 37, 49]. In the present study, PNFS was found to significantly reduce SBP and MBP in rats with MH in the first week of administration and sustain a stable reduction in blood pressure until 6 weeks of treatment. In addition, PNFS reduced face temperature and vertigo time and elevated grip strength in rats with MH. The effects of PNFS on the serum levels of TC, TG, HDL-c, and LDL-c in rats with MH were then further investigated and reflected the characteristics and beneficial effects of PNFS in treating MH. PNFS significantly reduced serum TC levels and PNFS-L significantly increased serum HDL-c levels in week 2 of administration; in week 4 of administration, PNFS significantly reduced the serum levels of TC and LDL-c, and PNFS-L significantly increased the serum levels of HDL-c. In addition, it reduced liver index, decreased hepatocyte swelling, reduced-fat vacuolation, and improved hepatic steatosis in rats with MH, indicating its benefits in preventing and treating MH.

C3 plays a crucial role in the pathogenesis of hypertension [50]. It was found to be highly expressed in the interstitial tissue of SHR and amplifies hypertension in SHR by activating KLF5 to secrete several enzymes and growth factors, such as ACE [51]. In addition, C3 increases the nuclear localization of LXRα via KLF5, which generates renin in the renal tubules [21], but this effect is inhibited in C3 gene–deficient SHR [52]. Previous studies have shown that *P. notoginseng* (Burkill) F. H. Chen total saponin can reduce serum C3 levels in patients with cerebral hemorrhage and inhibit the persistent elevation of C3 [53]. The present study found that PNFS not only downregulated the protein and mRNA expression of C3, KLF5, LXRα, and Renin in the kidneys but also decreased the plasma levels of renin, ACE, Ang II, and ALD in rats with MH. This was consistent with the previous results. Therefore, we conjectured that PNFS might retard the progression of MH by inhibiting RAAS through C3.

Endothelial impairment can occur by high renin and resulting high Ang II activity [54]. Vascular endothelial dysfunction is a phenotype directly related to cardiovascular diseases such as hypertension. ET-1, NO, TXB_2_, and PGI_2_ are vasoactive substances produced by vascular endothelial cells, reflecting vascular endothelial function. NO, and PGI_2_ are the main regulators of vascular tone. Endothelium-dependent vasodilatory dysfunction caused by various pathological factors can cause NO. Also, PGI_2_ synthesis-releasing properties are decreased, and blood pressure is increased [39]. ET-1 and TXB_2_ have vasoconstrictive effects, and their elevated levels are an important cause of hypertension [55, 56]. In the present study, vascular endothelial injury, increased the plasma levels of ET-1 and TXB_2_, and decreased levels of NO and PGI_2_ were found in rats with MH, consistent with previous results [4, 5]. PNFS corrected the imbalance of vasoactive substance levels in rats with MH and improved vascular histopathological changes (hyperplasia and disorganization of vascular smooth muscle cells, and misaligned and interrupted endothelial cells with detachment).

**Fig. 7:**
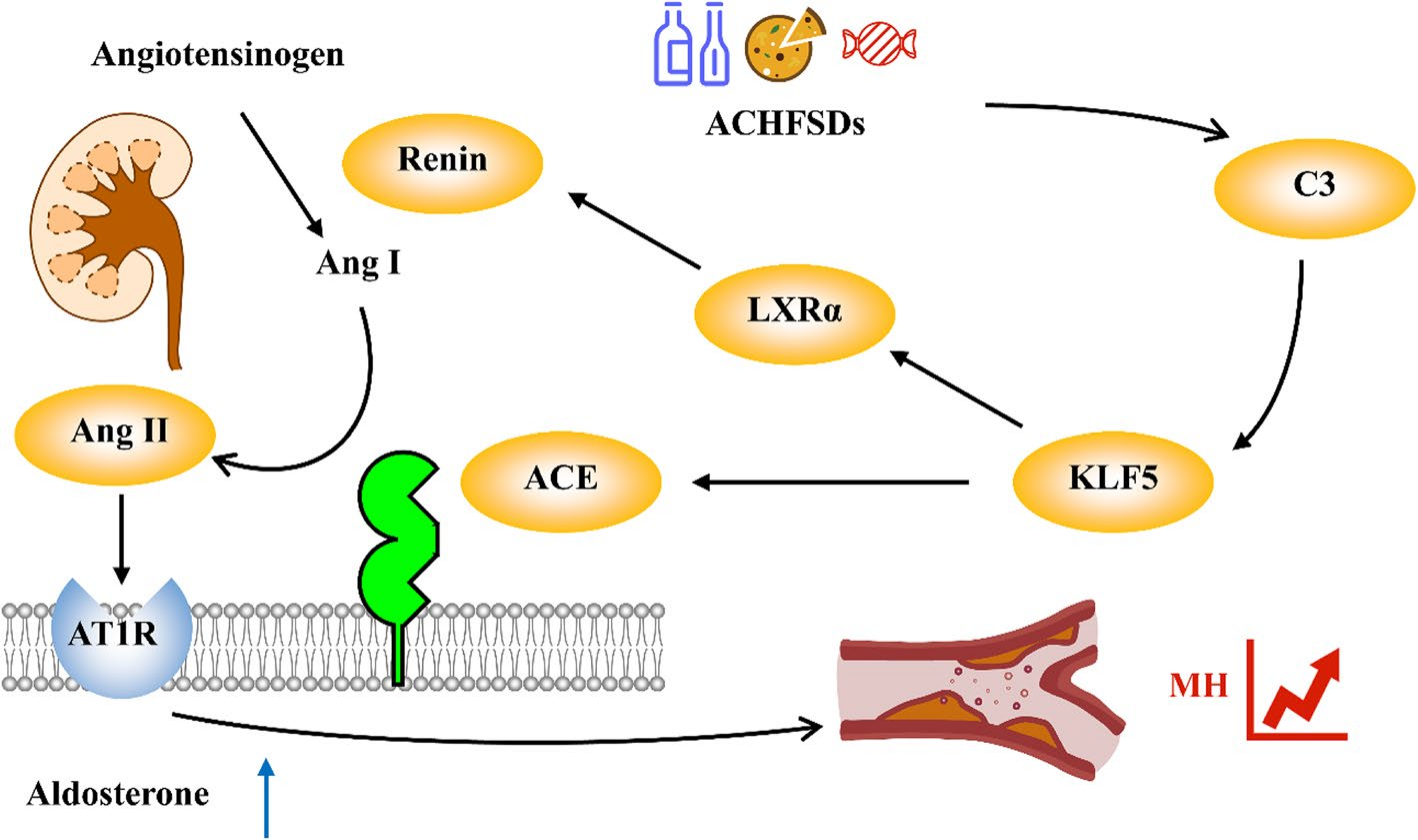
A summary diagram. This diagram shows the mechanism of over-eating alcohol, high fat, and sugar diets (ACHFSDs) leading to metabolic hypertension (MH)

In conclusion, ACHFSDs lead to an increase in C3 in the kidneys and then induce KLF5 to increase LXRα expression to stimulate Renin production. On the other hand, C3 also produces high levels of ACE through the activation of KLF5, which catalyzes the conversion of Ang I into Ang II, induces the activation of RAAS, disrupts vascular endothelial function, and thus induces MH (Fig. 7). PNFS reduces blood pressure in MH via this mechanism and lowers lipid levels, improving hepatic steatosis and lipid deposition. The present study only showed the effect of PNFS in lowering blood pressure in male metabolic hypertensive rats, giving some basis for the development and application of PNFS blood pressure-lowering related products. However, the effect of PNFS on female metabolic hypertensive rats still deserves further exploration.

## Conclusion

The antihypertensive effect and mechanism of PNFS on rats with MH were studied from three aspects. First, PNFS improved blood lipid levels and reduced hepatic steatosis. Second, PNFS modulated vascular-associated diastolic and systolic factors, including NO, PGI_2_, ET-1, and TXB_2_, and improved vascular endothelial function. Finally, PNFS suppressed RAAS activation and reduced blood pressure by regulating the expression of C3, KLF5, LXRα, and renin. This study provided a new focus on the mechanism of anti-MH in PNFS.

## Supplementary Information

**Additional file 1.**

## Data Availability

The datasets used and/or analyzed during the current study are available from the corresponding author on reasonable request.

## Abbreviations

MH: Metabolic Hypertension
ACHFSDs: Over-eating Alcohol, High Fat and Sugar Diets
PNFS: Panax Notoginseng (Burkill) F. H. Chen Flower Saponins
C3: Complement 3
Ang II: Angiotensin II
KLF5: Krüppel-like Factor 5
LXRα: Liver X Receptor α
RAAS: Renin-Angiotensin-Aldosterone System
SHR: Spontaneously Hypertensive Rats
SBP: Systolic Blood Pressure
TC: Total Cholesterol
TG: Triglyceride
LDL-c: Low-Density Lipoprotein cholesterol
HDL-c: High-Density Lipoprotein cholesterol
DBP: Diastolic Blood Pressure
ET-1: Endothelin 1
TXB_2_: Thromboxane B_2_
NO: Nitric Oxide
PGI_2_: Prostaglandin I_2_
MBP: Mean arterial Blood Pressure
ACE: Angiotensin-converting Enzyme
ALD: Aldosterone
H&E: Hematoxylin and Eosin
