# Correction: Beneficial effects of *Panax notoginseng* (Burkill) F. H. Chen flower saponins in rats with metabolic hypertension by inhibiting the activation of the renin–angiotensin–aldosterone system through complement 3

**DOI:** 10.1186/s12906-023-03893-1

**Published:** 2023-03-08

**Authors:** Qiqi Huang, Jie Su, Jie Xu, Huanhuan Yu, Xiaohu Jin, Yajun Wang, Meiqiu Yan, Jingjing Yu, Suhong Chen, Youhua Wang, Guiyuan Lv

**Affiliations:** 1 School of Pharmaceutical Sciences, Zhejiang Chinese Medical University, Hangzhou, China; 2 Collaborative Innovation Center of Yangtze River Delta Region Green Pharmaceuticals, Zhejiang University of Technology, Hangzhou, China; 3 Longhua Hospital, Shanghai University of Traditional Chinese Medicine, Shanghai, China

**Correction: BMC Complement Med Ther 23, 13 (2023)**

**https://doi.org/10.1186/s12906-022-03828-2**

Following the publication of the original article [1], it was noted that due to a typesetting error Dr. Guiyuan Lv was incorrectly affiliated to Longhua Hospital.

The correct affiliation for Dr. Guiyuan Lv should be School of Pharmaceutical Sciences, Zhejiang Chinese Medical University, Hangzhou, China.

The author group has been updated above and the original article [1] has been corrected.
