# Supplementary material for: Beneficial effects of Panax notoginseng (Burkill) F. H. Chen flower saponins in rats with metabolic hypertension by inhibiting the activation of the renin–angiotensin–aldosterone system through complement 3
Source: BMC Complement Med Ther. 2023 Jan 18;23:13. doi: 10.1186/s12906-022-03828-2 (PMC9847118; doi:10.1186/s12906-022-03828-2)
Supplement: Supplementary file 1 — Additional file 1. [file 12906_2022_3828_MOESM1_ESM.pdf]

Supplementary uncut and non-processed blots

Supplementary Original Western blots used for Fig.6A

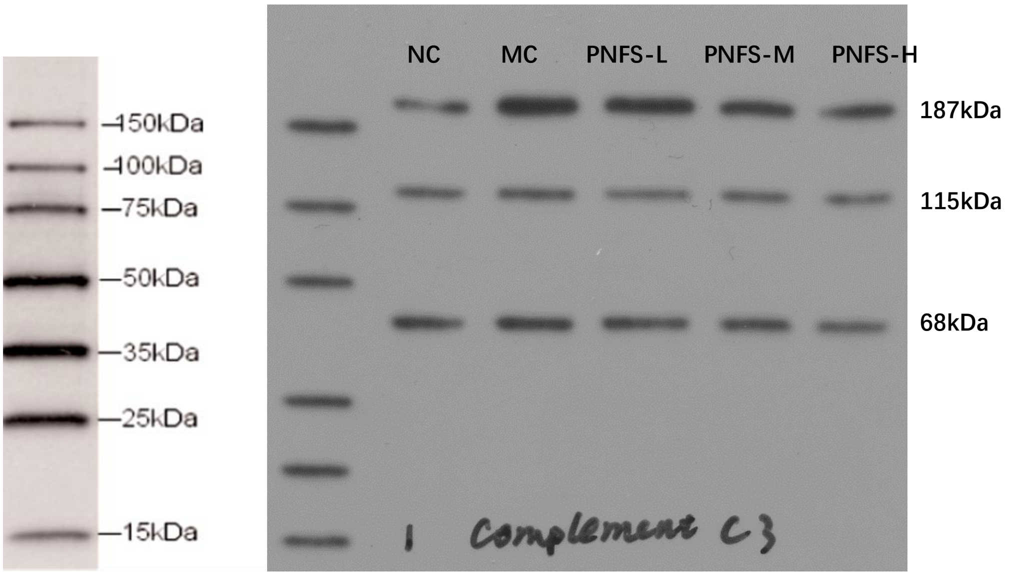

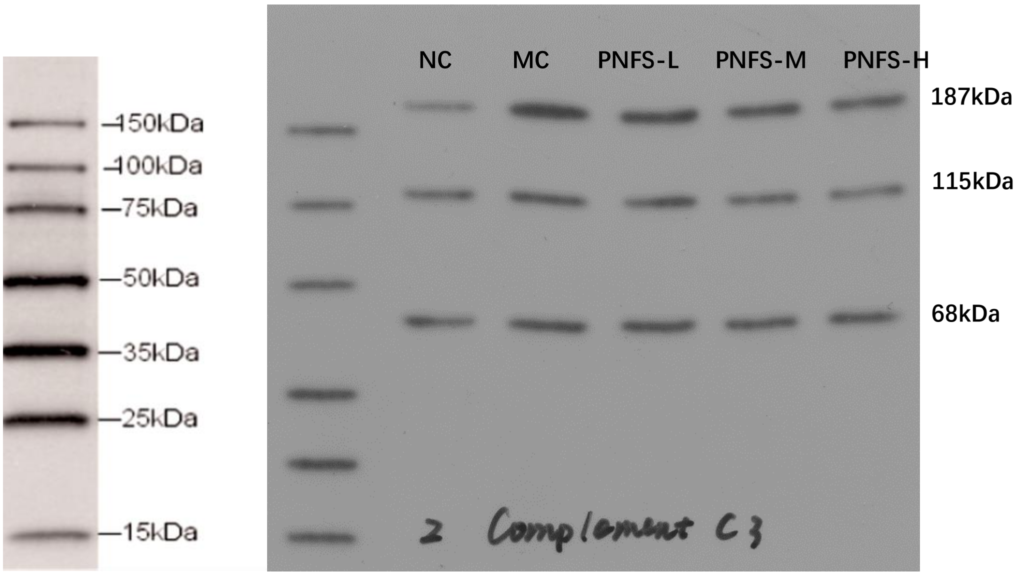

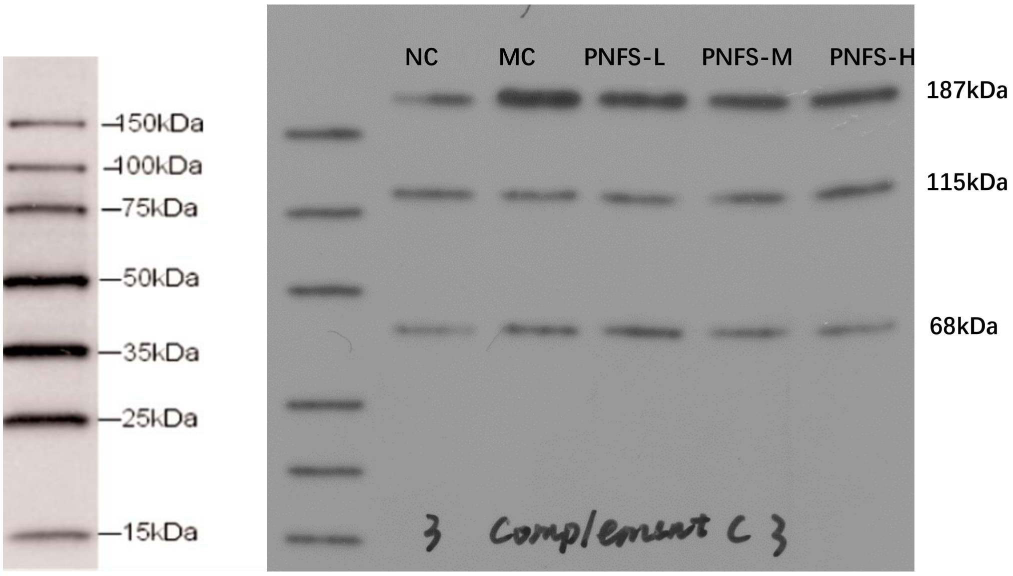

Fig.1 The uncut and and non-processed blot image of complement 3

Samples are labelled throughout as follows.

NC: normal control group

MC: model control group

PNFS-L: PNFS low dose group, 30.0 mg/kg

PNFS-M: PNFS middle dose group, 60.0 mg/kg

PNFS-H: PNFS high dose group, 120.0 mg/kg

This is the result of WB experiment with 3 samples in each group. All proteins are the same batch of samples. The protein as complement 3 bands are used for this manuscript.

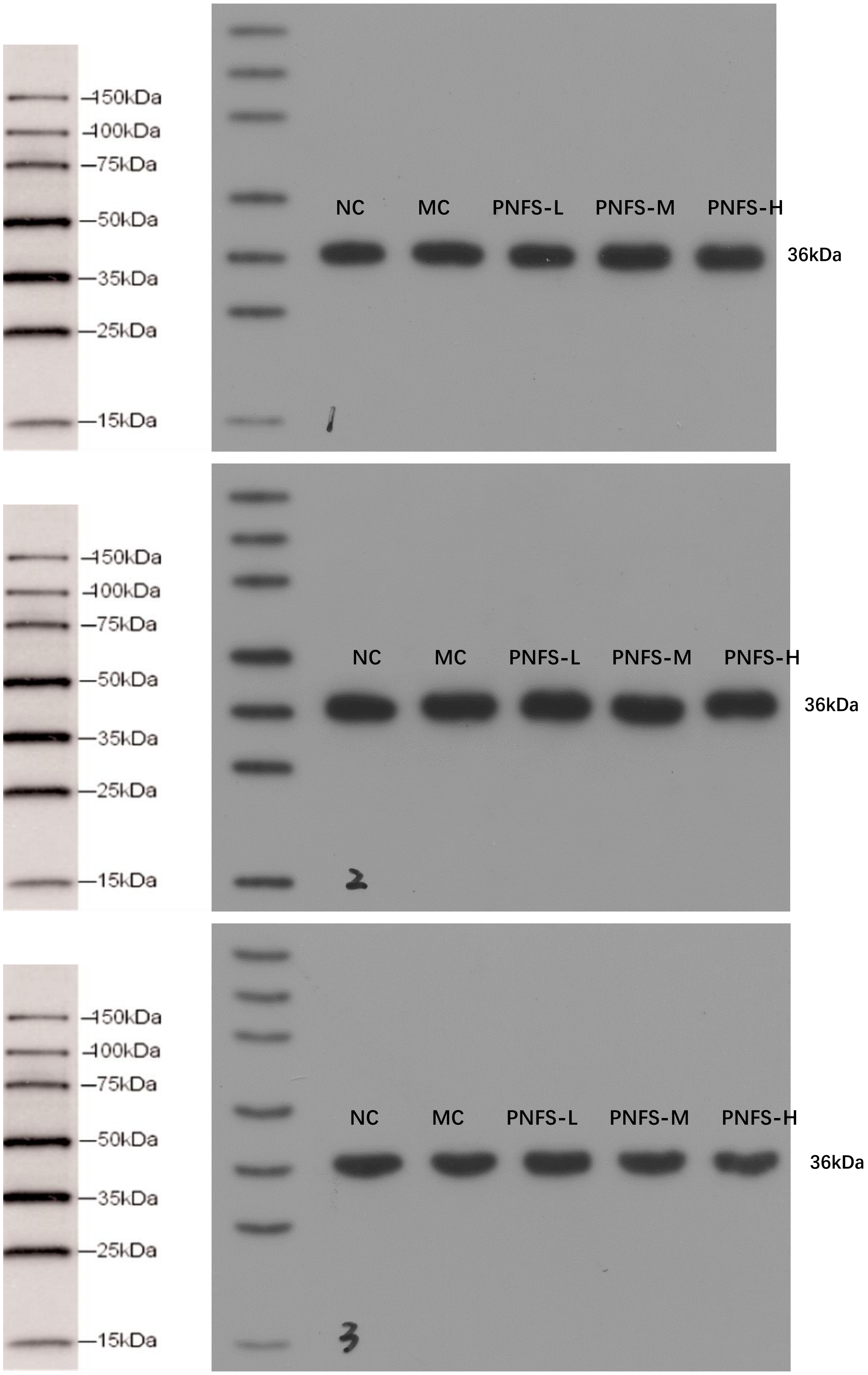
150kDa
100kDa
75kDa
50kDa
35kDa
25kDa
15kDa
NC
MC
PNFS-L
PNFS-M
PNFS-H
36kDa
1
2
3

Fig.2 The uncut and and non-processed blot image of GAPDH

Samples are labelled throughout as follows.

NC: normal control group

MC: model control group

PNFS-L: PNFS low dose group, 30.0 mg/kg

PNFS-M: PNFS middle dose group, 60.0 mg/kg

PNFS-H: PNFS high dose group, 120.0 mg/kg

This is the result of WB experiment with 3 samples in each group. All proteins are the same batch of samples. The protein as GAPDH bands are used for this manuscript.

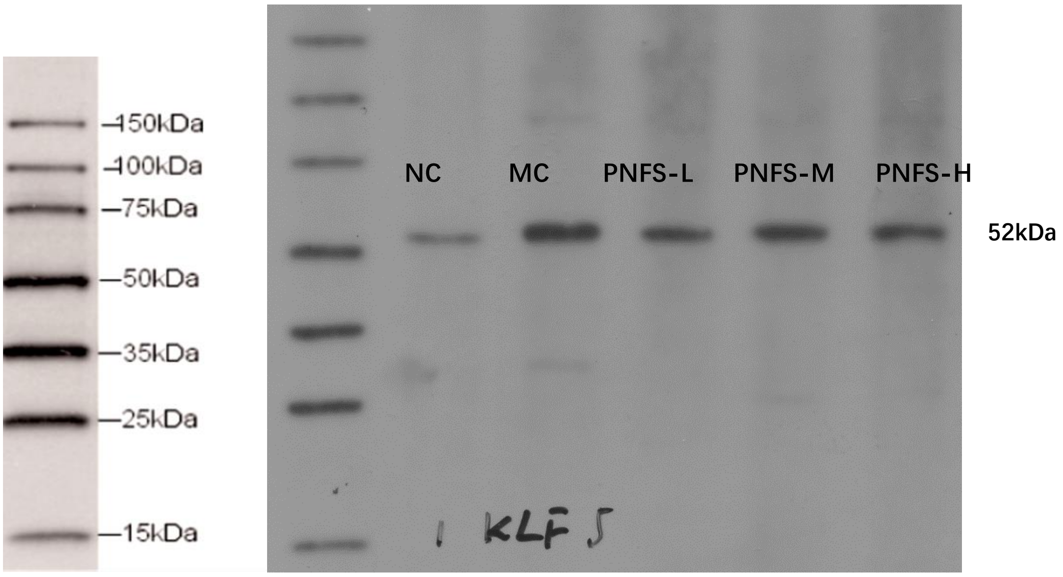
150kDa
100kDa
75kDa
50kDa
35kDa
25kDa
15kDa
NC
MC
PNFS-L
PNFS-M
PNFS-H
52kDa
1 KLF5

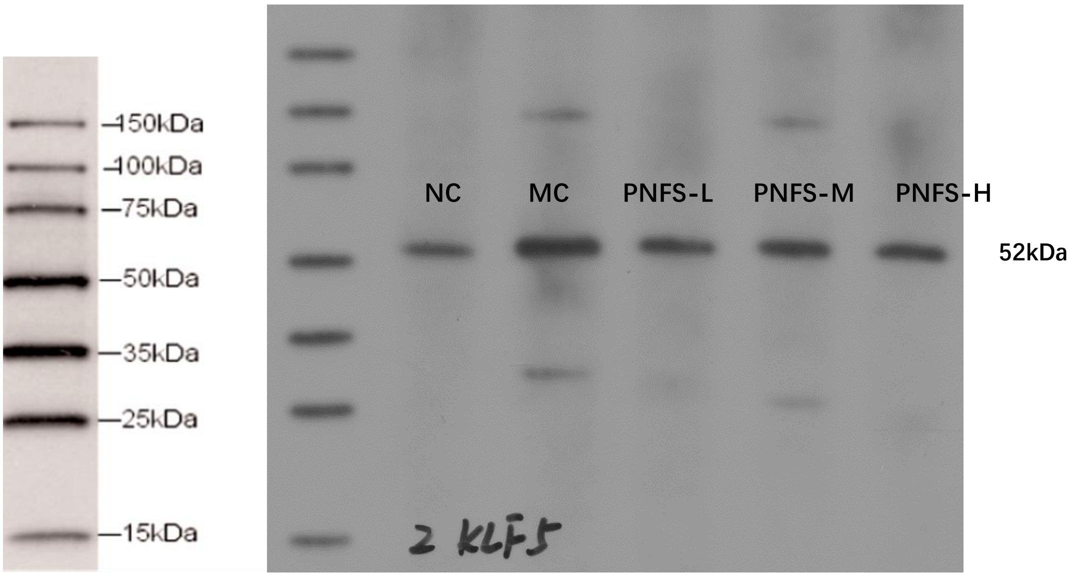
150kDa
100kDa
75kDa
50kDa
35kDa
25kDa
15kDa
NC
MC
PNFS-L
PNFS-M
PNFS-H
52kDa
2 KLF5

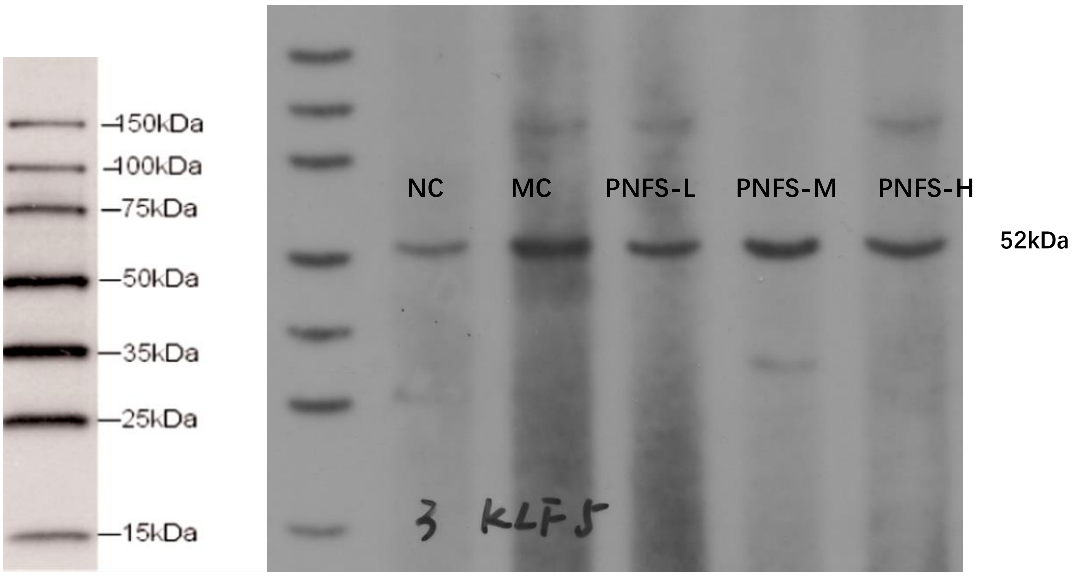
150kDa
100kDa
75kDa
50kDa
35kDa
25kDa
15kDa
NC
MC
PNFS-L
PNFS-M
PNFS-H
52kDa
3 KLF5

Fig.3 The uncut and and non-processed blot image of KLF5

Samples are labelled throughout as follows.

NC: normal control group

MC: model control group

PNFS-L: PNFS low dose group, 30.0 mg/kg

PNFS-M: PNFS middle dose group, 60.0 mg/kg

PNFS-H: PNFS high dose group, 120.0 mg/kg

This is the result of WB experiment with 3 samples in each group. All proteins are the same batch of samples. The protein as KLF5 bands are used for this manuscript.

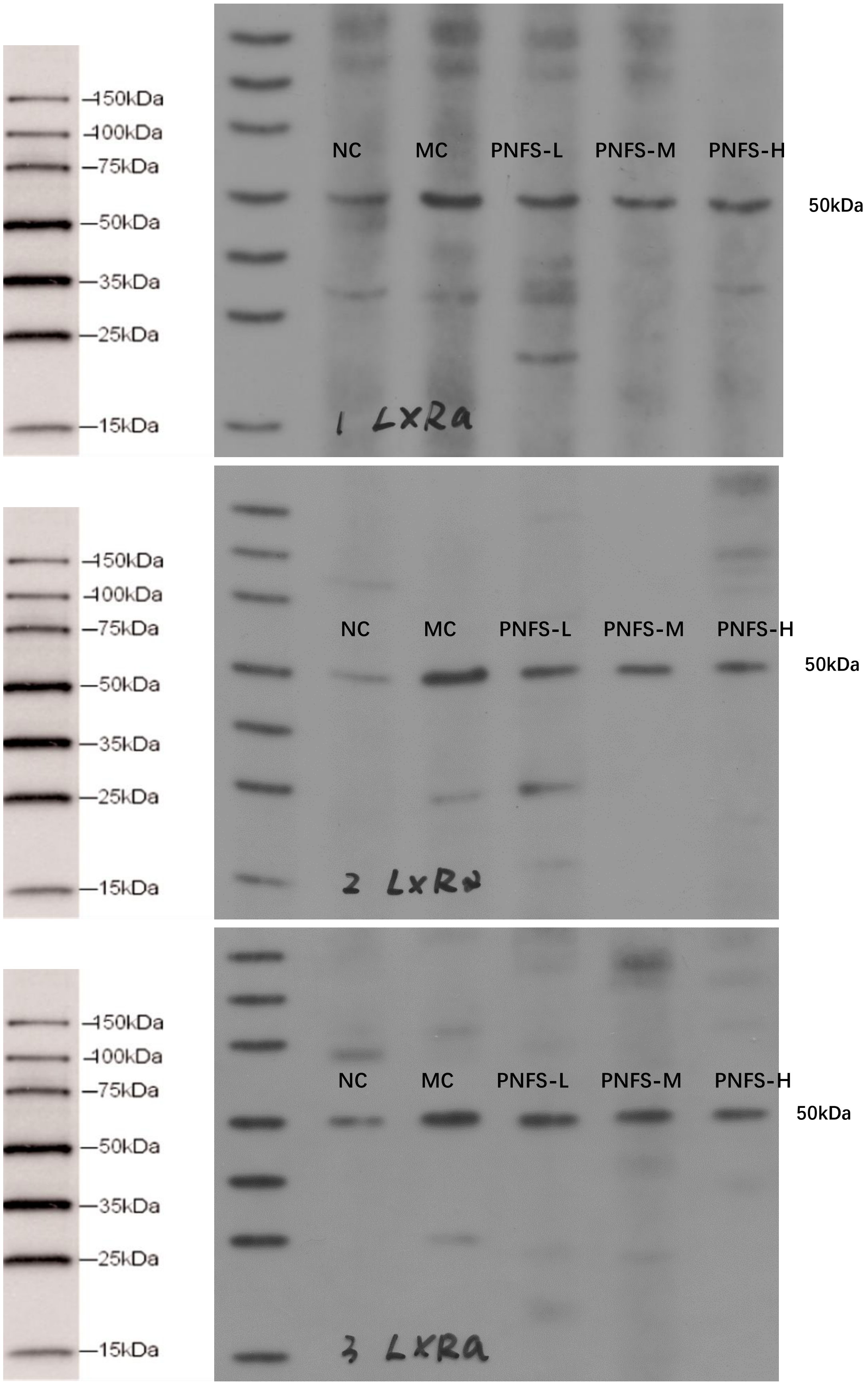

150kDa
100kDa
75kDa
50kDa
35kDa
25kDa
15kDa
NC
MC
PNFS-L
PNFS-M
PNFS-H
50kDa
1 LxRa
150kDa
100kDa
75kDa
50kDa
35kDa
25kDa
15kDa
NC
MC
PNFS-L
PNFS-M
PNFS-H
50kDa
2 LxR
150kDa
100kDa
75kDa
50kDa
35kDa
25kDa
15kDa
NC
MC
PNFS-L
PNFS-M
PNFS-H
50kDa
3 LxRa

Fig.4 The uncut and and non-processed blot image of LXRα

Samples are labelled throughout as follows.

NC: normal control group

MC: model control group

PNFS-L: PNFS low dose group, 30.0 mg/kg

PNFS-M: PNFS middle dose group, 60.0 mg/kg

PNFS-H: PNFS high dose group, 120.0 mg/kg

This is the result of WB experiment with 3 samples in each group. All proteins are the same batch of samples. The protein as LXRα bands are used for this manuscript.

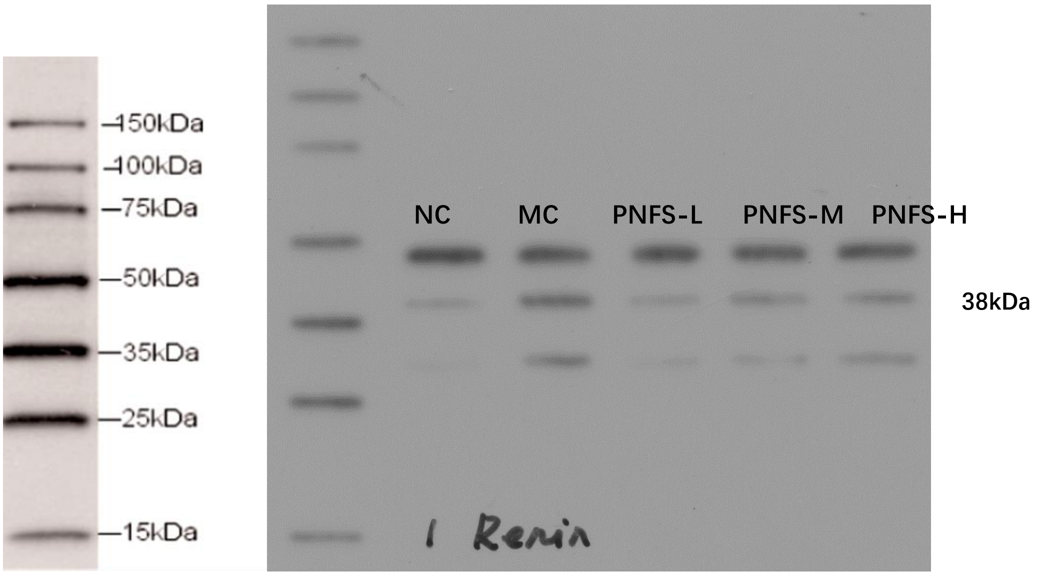
150kDa
100kDa
75kDa
50kDa
35kDa
25kDa
15kDa
NC
MC
PNFS-L
PNFS-M
PNFS-H
38kDa
1 Renin

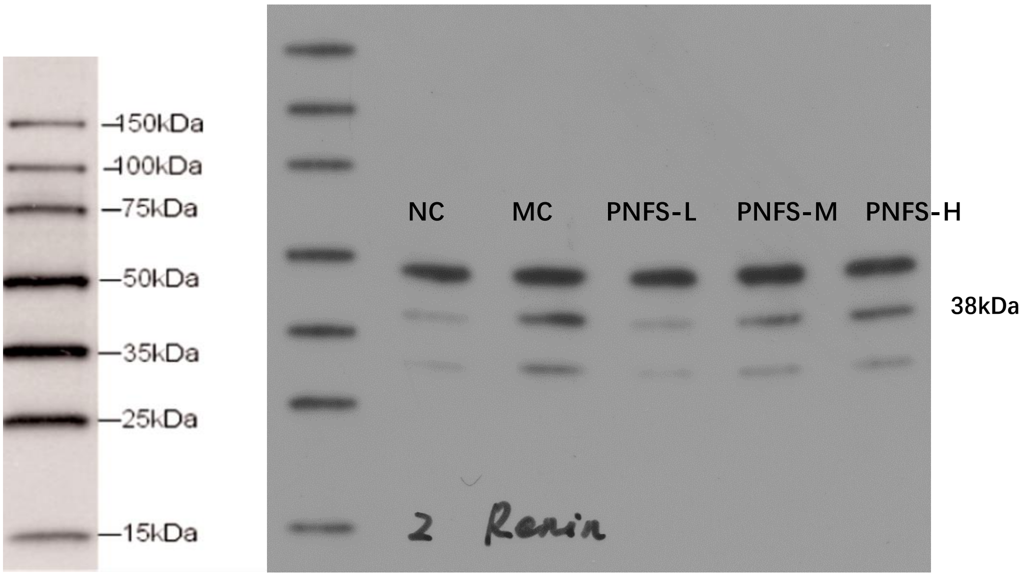
150kDa
100kDa
75kDa
50kDa
35kDa
25kDa
15kDa
NC
MC
PNFS-L
PNFS-M
PNFS-H
38kDa
2 Renin

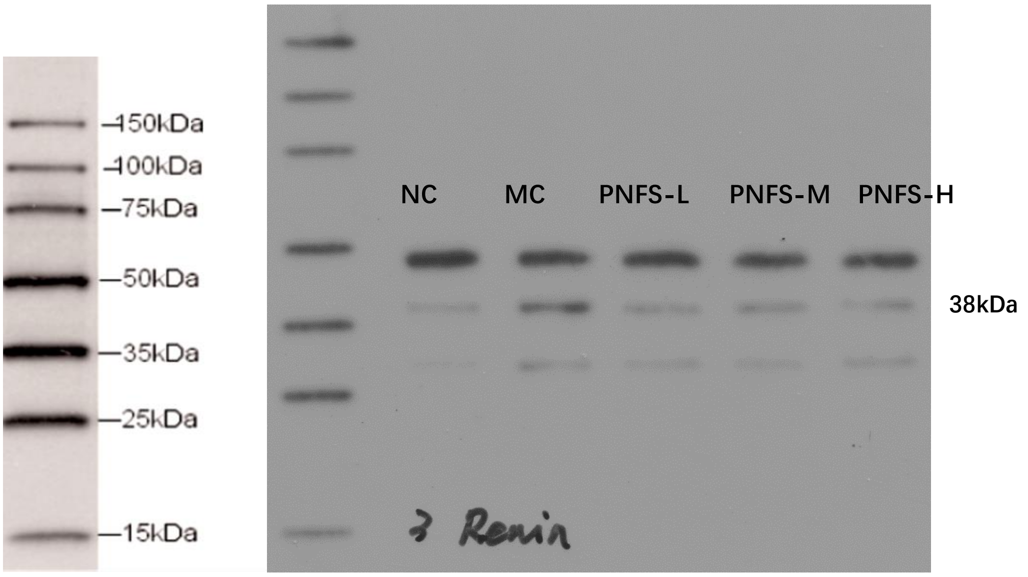
150kDa
100kDa
75kDa
50kDa
35kDa
25kDa
15kDa
NC
MC
PNFS-L
PNFS-M
PNFS-H
38kDa
3 Renin

Fig.5 The uncut and and non-processed blot image of Renin

Samples are labelled throughout as follows.

NC: normal control group

MC: model control group

PNFS-L: PNFS low dose group, 30.0 mg/kg

PNFS-M: PNFS middle dose group, 60.0 mg/kg

PNFS-H: PNFS high dose group, 120.0 mg/kg

This is the result of WB experiment with 3 samples in each group. All proteins are the same batch of samples. The protein as Renin bands are used for this manuscript.
